# PGC-1α dictates endothelial function through regulation of eNOS expression

**DOI:** 10.1038/srep38210

**Published:** 2016-12-02

**Authors:** Siobhan M. Craige, Swenja Kröller-Schön, Chunying Li, Shashi Kant, Shenghe Cai, Kai Chen, Mayur M. Contractor, Yongmei Pei, Eberhard Schulz, John F. Keaney

**Affiliations:** 1 Division of Cardiovascular Medicine, Department of Medicine, University of Massachusetts Medical School, Worcester, MA, USA; 2 Department of Cardiology Medizinische Klinik und Poliklinik Universitätsmedizin Mainz, Mainz, Germany; 3 University of Connecticut Health Center, Farmington, CT, USA.

## Abstract

Endothelial dysfunction is a characteristic of many vascular related diseases such as hypertension. Peroxisome proliferator activated receptor gamma, coactivator 1α (PGC-1α) is a unique stress sensor that largely acts to promote adaptive responses. Therefore, we sought to define the role of endothelial PGC-1α in vascular function using mice with endothelial specific loss of function (PGC-1α EC KO) and endothelial specific gain of function (PGC-1α EC TG). Here we report that endothelial PGC-1α is suppressed in angiotensin-II (ATII)-induced hypertension. Deletion of endothelial PGC-1α sensitized mice to endothelial dysfunction and hypertension in response to ATII, whereas PGC-1α EC TG mice were protected. Mechanistically, PGC-1α promotes eNOS expression and activity, which is necessary for protection from ATII-induced dysfunction as mice either treated with an eNOS inhibitor (LNAME) or lacking eNOS were no longer responsive to transgenic endothelial PGC-1α expression. Finally, we determined that the orphan nuclear receptor, estrogen related receptor α (ERRα) is required to coordinate the PGC-1α -induced eNOS expression. In conclusion, endothelial PGC-1α expression protects from vascular dysfunction by promoting NO• bioactivity through ERRα induced expression of eNOS.

Hypertension is the most prevalent risk factor for vascular disease worldwide, with expectations that this condition will impact up to 1.56 billion people by the year 2025^1^. Patients with hypertension are at increased risk of heart attack and stroke, two major sources of cardiovascular morbidity and mortality. Among the sequelae of hypertension, endothelial dysfunction, characterized by compromised nitric oxide (NO•) bioavailability^2^,^3^, is particularly prominent. Indeed, endothelial dysfunction has proven a powerful predictor of cardiovascular events^4^. Thus, there is considerable interest in developing strategies to improve endothelial function and NO• bioactivity as a means to ameliorate the consequences of hypertension, including cardiovascular disease.

Metabolic pathways have garnered increasing attention as being relevant to vascular disease. In this regard, caloric restriction and exercise – two conditions that alter the balance between energy storage and utilization, have emerged as strategies to mitigate chronic disease states, such as hypertension and its impact on the vasculature. Both caloric restriction and exercise activate the energy-sensitive enzyme, AMP-activated protein kinase (AMPK), that can limit the development of endothelial dysfunction through decreased vascular reactive oxygen species (ROS) and improved NO• bioavailability^5^. One important downstream target of AMPK is peroxisome proliferator activated receptor gamma, coactivator 1α (PGC-1α)^6^, a transcriptional coactivator important for the regulation of both mitochondrial and cellular genes involved in metabolism and stress adaptation^7^,^8^. PGC-1α expression is decreased in aging^9^ and may be important for the aging associated decline of vascular function^10^. *In vitro*, endothelial PGC-1α demonstrates improved ROS detoxification and enhanced endothelial resistance to cellular injury^11^,^12^. However, the role of PGC-1α on the endothelium *in vivo* is not yet clear. Thus, we probed the impact of endothelial specific PGC-1α manipulation on mouse models of endothelial dysfunction and hypertension.

## Results

### Loss of endothelial PGC-1α impairs endothelial NO• bioactivity

**Figure 1:**
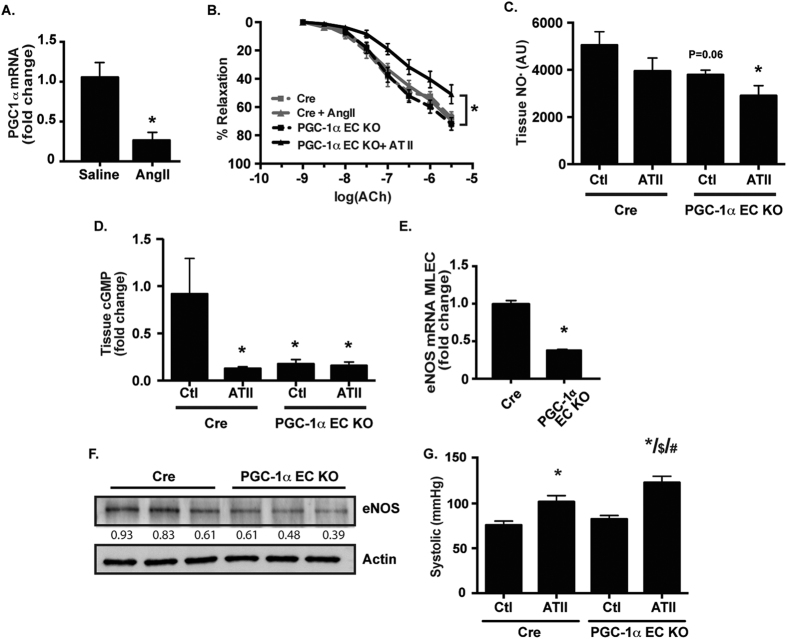
Loss of endothelial PGC-1α leads to reduced NO• bioactivity. (**A**) WT mice received a pressor dose of Angiotensin II (ATII; 1 mg/kg; 7 days). Mouse lung endothelial cells (MLECs) were harvested and PGC-1α mRNA expression measured (N = 4/group, *P < 0.05 vs. saline). Endothelial specific PGC-1α knock out (PGC-1α EC KO) mice and control EC Cre+ mice (Cre) were treated with a subpressor dose of ATII (0. 5 mg/kg; 7 days) and endothelial function estimated as (**B**) aortic isometric force in response to acetylcholine (Ach; N = 10–15/group, *P < 0.05 vs. Cre without ATII), (**C**) vessel NO• levels (N = 6/group, *P < 0.05 vs. Cre Ctl) and (**D**) tissue cGMP levels (N = 12/group *P < 0.05 vs. Cre Ctl). MLECs harvested from PGC-1α EC KO mice were used to measure eNOS (**E**) mRNA (N = 4–6/group, *P < 0.05 vs. Cre) or (**F**) protein (N = 3/group) expression. (**G**) Cre and PGC-1α EC KO mice were treated with a pressor dose of ATII (as in A) and systolic blood pressure was recorded (N = 4–8/group; *P < 0.05 vs Cre Ctl; ^$^*P* < 0.05 vs Cre ATII; ^#^*P* < 0.05 vs PGC-1α EC KO Ctl). Numbers below immunoblots represent densitometry relative to actin.

Previously we have demonstrated that the endothelium is modulated by mediators of mitochondrial function such as AMP kinase^5^ and uncoupling protein-2^13^. As PGC-1α is well-established to mediate mitochondrial biogenesis and metabolic stress adaptation in other cell types^6^, we examined PGC-1α expression in endothelial cells from wild-type (WT) mice treated with a pressor dose (i.e, a dose known to induce endothelial dysfunction in WT mice) of angiotensin II (ATII). We observed a significant decrease in endothelial PGC-1α expression (Fig. 1A). These data suggest that suppression of endothelial PGC-1α expression may be important in promoting endothelial dysfunction in response to ATII.

To examine if loss of endothelial PGC-1α primes the endothelium for dysfunction, we created endothelial-specific loss of function (PGC-1α EC KO) mice which demonstrated a significant reduction in PGC-1α expression (Supplementary Figure 1A and B). We then examined endothelial function in response to ATII at a subpressor dose (i.e, a dose that does not significantly compromise endothelial function in WT mice). We found that loss of endothelial PGC-1α significantly sensitized the endothelium to dysfunction induced by ATII (Fig. 1B). Similarly, tissue NO• abundance (Fig. 1C) and eNOS expression (Supplementary Figure 1B and 1C) was decreased in the PGC-1α EC KO mice treated with ATII. Tissue cGMP, a downstream read out of bioactive NO•, was attenuated in the PGC-1α EC KO mice (Fig. 1D). In addition, endothelial cells from PGC-1α EC KO mice exhibited decreased eNOS mRNA (Fig. 1E) and protein (Fig. 1F) expression. These data suggest that loss of PGC-1α leads to compromised endothelial function, through decreases in eNOS expression and NO• bioactivity. Finally, we tested a pressor ATII dose (Fig. 1G) and found that the PGC-1α EC KO mice had significantly increased blood pressure compared to the Cre control mice.

### Endothelial PGC-1α protects from angiotensin II-induced hypertension and dysfunction *in vivo*

**Figure 2:**
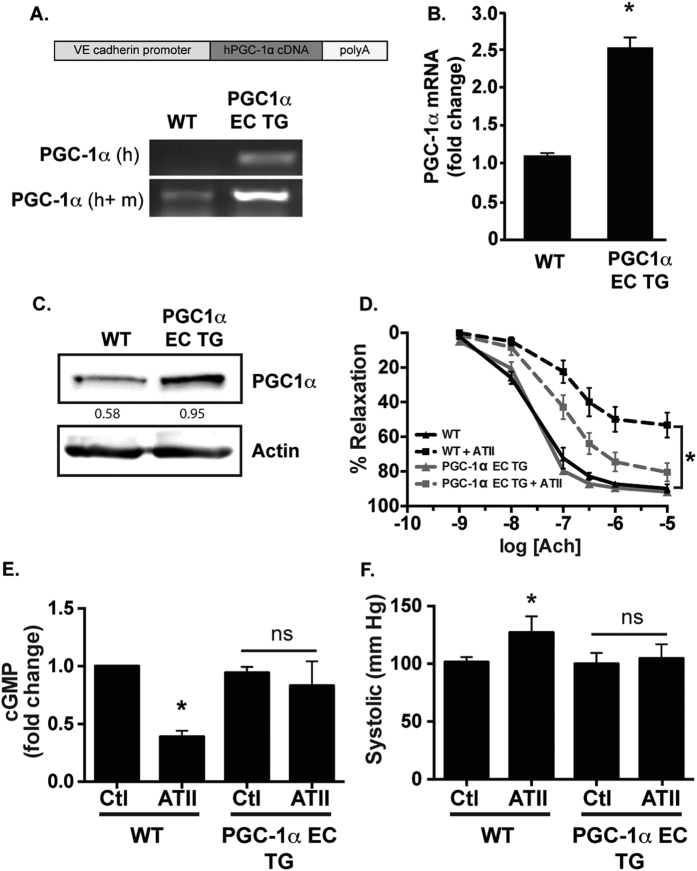
Endothelial PGC-1α overexpression protects from endothelial dysfunction. (**A**) PGC-1α endothelial specific transgene construct with representative expression of human (h) and mouse (m) PGC-1α mRNA in MLECs from the indicated genotype. (**B**) MLEC mRNA quantification (N = 3, *P < 0.05) (**C**) MLEC PGC-1α protein expression from the indicated mouse genotype. WT and PGC-1α EC TG mice were infused with a pressor dose of ATII (1 mg/kg; 7 days) for 7 days and examined for: (**D**) Aortic relaxation to Ach (N = 7–10/group; *P < 0.05 vs. WT + ATII), (**E**) cGMP content in aortic segments (N = 4/group; *P < 0.05 vs. WT Ctl) and (**F**) blood pressure of WT and PGC-1α EC TG mice treated with ATII (N = 9–11/group; *P < 0.05 vs WT Ctl).

To determine if upregulation of endothelial PGC-1α impacts NO• bioactivity, we created transgenic mice with constitutive human PGC-1α expression under the control of the vascular endothelial cadherin promoter (PGC-1α EC TG; Fig. 2A). These mice demonstrated increased endothelial PGC-1α expression (Fig. 2B and C). After a pressor dose of ATII, relaxation in response to acetylcholine (Ach) was attenuated in WT aorta, but largely preserved in vessels from PGC-1α EC TG mice (Fig. 2D). Similarly, NO• bioactivity after ATII treatment was preserved in PGC-1α EC TG mice as demonstrated by aortic tissue cGMP levels (Fig. 2E). We then tested the impact of preserved endothelial NO• bioactivity on ATII-induced hypertension. In WT mice, ATII infusion caused a significant increase in blood pressure as previously reported (Fig. 2F)^14^. This increase was prevented in PGC-1α EC TG mice (Fig. 2F). Together, these data confirm that augmentation of endothelial PGC-1α expression *in vivo* results in protection from endothelial dysfunction.

### PGC-1α expression protects the endothelium through enhanced eNOS expression and NO• bioactivity

**Figure 3:**
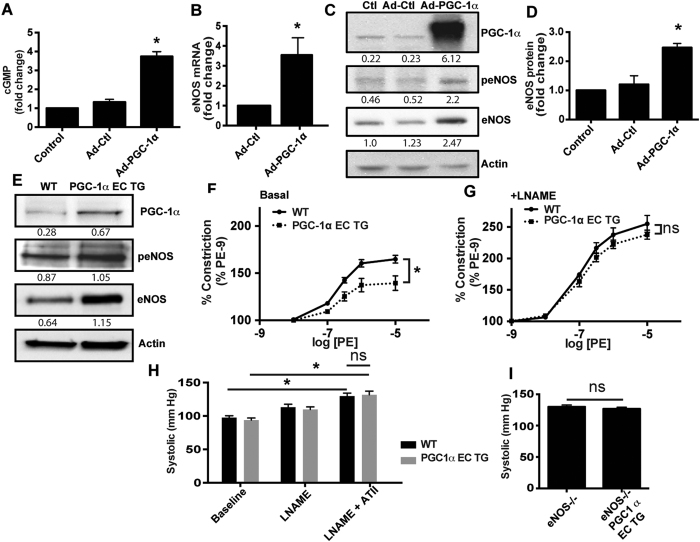
PGC-1α upregulates eNOS expression and limits hypertension-induced endothelial dysfunction. Human aortic endothelial cells (HAECs) were transfected or not with adenoviral (Ad) vectors containing β-galactosidase (Ctl) or PGC-1α for 24 h and assessed for (**A**) cGMP (N = 3), eNOS (**B**) mRNA (N = 3) and (**C**,**D**) protein; *P < 0.05 vs. Ctl. (**E**) Immunoblots for expression of the indicated proteins in MLECs from WT and PGC-1α EC TG mice (peNOS = phospho-Ser1177). Aortic segment contraction to phenylephrine (PE) in the absence (**F**) or presence (**G**) of 300 μM LNAME (N = 5–7/group; *P < 0.05 vs. WT). (**H**) Systolic blood pressure in WT and PGC-1α EC TG mice treated with LNAME (0.5 g/L in drinking water for 7 days) with or without pressor doses of ATII for 7 days (N = 8; *P < 0.05). (**I**) Systolic blood pressure in response to ATII as a function of the indicated genotype (N = 16–18/grp).

It is known *in vitro* that PGC-1α can protect the endothelium from ROS-induced damage^11^,^12^. However, the impact of PGC1α on endothelial NO• generation is not known *in vivo*. Thus, we sought to determine if PGC-1α overexpression influences NO• production and eNOS expression. In human aortic endothelial cells (HAECs), forced adenoviral overexpression of PGC-1α (Ad- PGC-1α) was associated with enhanced NO• bioactivity assessed through cGMP production (Fig. 3A) coincident with a significant increase in eNOS mRNA (Fig. 3B) and protein expression (Fig. 3C and D). We observed a modest increase in phosphorylated eNOS (Ser1177), likely due to the total eNOS increase (Fig. 3D). Consistent with these findings, there was increased eNOS expression in endothelial cells from the PGC-1α EC TG mice (Fig. 3E). Aortic segments from PGC-1α EC TG mice exhibited reduced constriction in response to phenylephrine (Fig. 3F), consistent with increased basal NO• bioactivity. This response was specific to eNOS as it was abrogated by the eNOS inhibitor, L-Nitroarginine Methyl Ester (LNAME; 300 μM; Fig. 3G). We examined the ability of endothelial PGC-1α expression to prevent ATII - induced hypertension in a prospective study where the WT and PGC-1α EC TG cohorts were administered LNAME (0.5 g/L) in the drinking water for 7 days followed by 7 days of LNAME + ATII. Results from this experiment demonstrated that eNOS was necessary for PGC-1α protection from hypertension (Fig. 3H). The requirement for eNOS was further tested by breeding the PGC-1α EC TG mice onto the eNOS-null background (eNOS^−/−^). Endothelial nitric oxide synthase null mice are spontaneously hypertensive and exhibit endothelial dysfunction^15^. In these mice, the endothelial specific expression of PGC-1α was not protective from spontaneous hypertension (Fig. 3I). These data confirm that eNOS is a key contributor in mediating PGC-1α protection of the endothelium.

### PGC-1α promotes eNOS expression through ERRα

**Figure 4:**
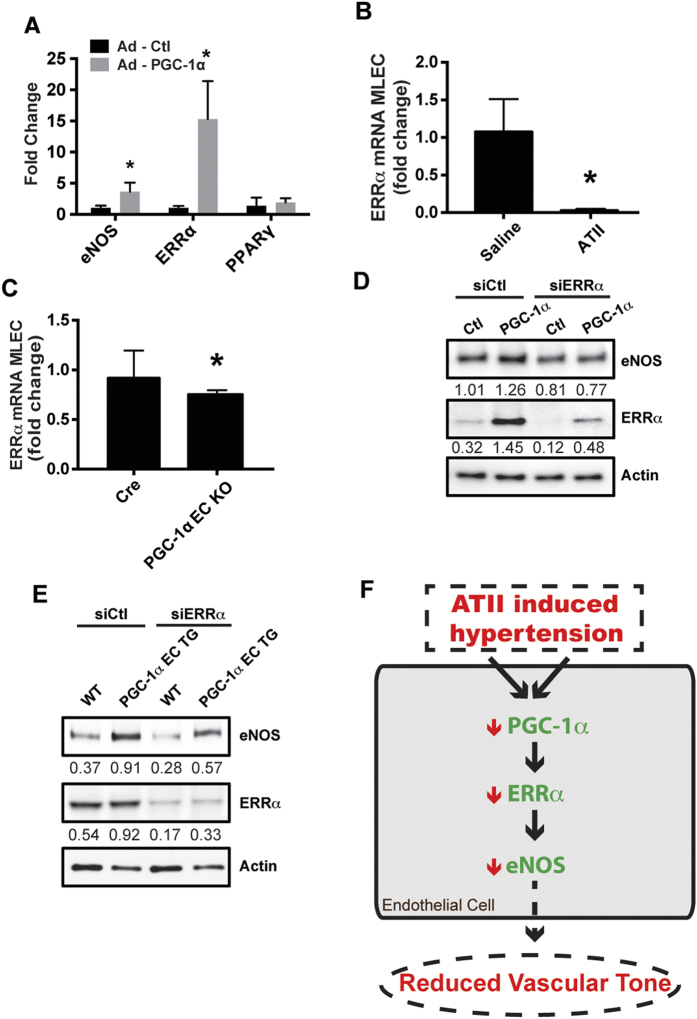
ERRα is required for PGC-1α dependent eNOS expression. (**A**) HAECs were transfected with adenoviral vectors expressing β-galactosidase (Ad-Ctl) or PGC-1α (Ad- PGC-1α) and mRNA expression of the indicated genes determined by RT-PCR (N = 3, *P < 0.05). (**B**) ERRα mRNA in MLEC from mice treated with saline or ATII (1 mg/kg; 7days; N = 4/group). (**C**) ERRα mRNA in MLEC from Cre or PGC-1α EC KO mice (N = 3; *P < 0.05). (**D**) HUVEC were treated with adenovirus expressing irrelevant siRNA (siCtl) or ERRα siRNA (siERRα) for 48 h followed by treatment with Ad-GFP or Ad-PGC-1α for 24 h. (**E**) MLEC from WT and PGC-1α EC TG mice were treated with adenovirus as in **D** (siCtl or siERRα). Cell lysates were then immunoblotted for the indicated proteins. (**F**) Schematic of ATII-induced hypertension and PGC-1α in the endothelium.

One common mechanism of eNOS activation and upregulation is via the serine/threonine kinase, Akt^16^,^17^, therefore, we examined Akt expression and activation. In our studies, PGC-1α did not increase Akt activation or expression (Supplementary Figure 2A). Another mechanism of eNOS activation is through reactive oxygen species (ROS) as we and others have previously demonstrated that ROS can increase eNOS activity and expression^18^,^19^. However, we found decreased ROS production in endothelial cells from PGC-1α EC TG mice compared to WT mice (Supplementary Figure 2B). Thus, we turned our attention to well-known PGC-1α targets such as Estrogen Related Receptor α (ERRα and Peroxisome Proliferator-Activated Receptor γ (PPARγ), that can modify eNOS expression or activity^20^. Forced expression of PGC-1α in HAECs produced no change in PPARγ expression, but a significant increase in ERRα (Fig. 4A). Endothelial cells harvested from WT mice treated with ATII demonstrated suppressed ERRα expression (Fig. 4B), reminiscent of the effect on PGC-1α (Fig. 1A). Furthermore, PGC-1α EC KO endothelial cells exhibit decreased ERRα expression (Fig. 4C), demonstrating that in endothelial cells, ERRα expression closely mimics the expression pattern of PGC-1α. We then investigated whether ERRα was necessary for the increased expression of eNOS. As ERRα has been shown to induce eNOS expression^20^, we used siRNA to knock down ERRα in HUVECs (Fig. 4D) and endothelial cells from our PGC-1α EC TG mice (Fig. 4E). In both of these cell types, PGC-1α mediated increases in eNOS expression were attenuated as demonstrated in summary (Fig. 4F).

## Discussion

The data presented here indicate that PGC-1α expression plays an important role in the pathophysiology of angiotensin II-induced hypertension. Downregulation of PGC-1α was observed during ATII-infusion and loss of PGC-1α facilitated the development of endothelial cell dysfunction, whereas persistent endothelial PGC-1α expression attenuated the response to ATII. Of particular note, we identified a new role for PGC-1α in maintaining eNOS expression: PGC-1α loss of function was associated with a reduction in eNOS expression and, conversely, PGC-1α gain of function increased basal eNOS expression. Furthermore, PGC-1α required ERRα to enhance eNOS expression. Collectively, these data indicate that PGC-1α is a key novel determinant of endothelial cell eNOS expression and, as a consequence, NO• bioactivity.

Although previous data has linked PGC-1α to nitric oxide dependent responses, generally the literature has been focused on the effects of NO• on PGC-1α, rather than vice versa as we describe here. For example, NO• has been shown to upregulate PGC-1α and stimulate mitochondrial biogenesis^21^. Likewise, caloric restriction, a state associated with increased longevity, causes mitochondrial biogenesis and PGC-1α upregulation in an eNOS-dependent manner^22^. The data presented here suggest a bidirectional relationship between PGC-1α and NO•, with the former being required to maintain normal eNOS expression in the endothelium.

Furthermore, we demonstrate that PGC-1α expression dictates endothelial function in the context of angiotensin II-induced hypertension, a disease model characterized by impaired NO• bioactivity and increased vascular ROS production^23^. With regard to the latter, there are several PGC-1α-dependent genes known to coordinate antioxidant gene regulation, including SOD2, Prx3, Prx5, Trx 2, and catalase^12^. These data, combined with observations that PGC-1α protects the endothelium from ROS-mediated damage^11^,^12^,^24^, suggest that one potential mechanism for our observations may also be the antioxidant program promoted by PGC-1α. This idea is supported by observations that manipulation of vascular ROS scavenging are associated with improved NO• bioactivity and preservation of endothelial function^25^. However, the relative importance to the endothelium of PGC-1α action on ROS scavenging versus its effect on eNOS expression remains to be determined. Nevertheless, our data indicate that PGC-1α cannot significantly impact the consequences of ATII in the absence of eNOS.

In addition to ROS scavenging, PGC-1α expression has been implicated in protection from inflammation. For example, PGC-1α overexpression results in decreased endothelial inflammation in response to tumor necrosis factor-α^26^ PGC-1α deficient mice bred onto the ApoE^−/−^ background displayed increased inflammatory markers in plaques^27^. As ATII-induced hypertension is associated with a vascular inflammatory response with increased endothelial inflammatory gene expression^28^, one might speculate that endothelial PGC-1α manipulation may impact vascular inflammatory responses.

While, multiple studies in endothelial cells have demonstrated protection from ROS^12^,^22^, cell death^29^, and inflammation^26^, in conditions of high glucose, PGC-1α may have a detrimental effect and lead to impaired endothelial function. For instance, under diabetic conditions, PGC-1α overexpression led to decreased angiogenesis, whereas PGC-1α loss of function improved endothelial angiogenesis^30^. Therefore, endothelial PGC-1α could potentially serve multiple contextual roles wherein conditions of hypertension, lead to improved eNOS bioavailability, but in conditions of hyperglycemia, endothelial angiogenesis is impeded. Further investigation will be required for a complete picture as to how PGC-1α contributes to endothelial phenotype in health and disease.

The data presented here demonstrate a novel paradigm wherein PGC-1α induces expression of ERRα, which is then necessary to enhance eNOS expression (Fig. 4D,E). In our study, endothelial ERRα expression mirrored PGC-1α expression. Although it was known that the eNOS promoter contains an ERRα binding site^20^, we have now elucidated a functional connection to eNOS *in vivo*. The link between ERRα and PGC-1α is consistent with studies in mice lacking ERRα, that phenocopy the heart failure seen in PGC-1α knockout mice^31^. However, studies with endothelial-specific ERRα manipulation *in vivo* will be needed to further clarify its role in vascular function.

In summary, the work presented here links endothelial PGC-1α to NO• bioactivity via eNOS regulation. This indicates that endothelial PGC-1α expression may be an important determinant of vascular health (Fig. 4F) as it is protective from endothelial dysfunction in response to ATII. It will be of particular interest to determine if the molecular pathway described here impacts other critical processes involved in endothelial dysfunction, such as mitochondrial function, metabolism, and inflammation.

## Methods

### Materials

Antibodies: PGC-1α (Abcam); phospho-Ser1177 eNOS (Upstate Biotechnology); eNOS (BD Transduction); ERRα (Abcam); β-actin (Sigma). Micro-osmotic pumps were purchased from DURECT corporation (Cupertino, CA). Other chemicals were obtained from Sigma.

### Generation of endothelial specific PGC-1α manipulation in mice

Human PGC-1α cDNA (Origene, Rockville, MD) was linearized and inserted into pBSmVELacZ (Obtained from Kenneth Walsh, Ph.D. Boston University, Boston, MA) to replace the LacZ open reading frame through the NotI site to produce human PGC-1α expression under the control of the mouse vascular endothelial cadherin promoter (VE-Cad; Fig. 2A). The resulting construct was microinjected into fertilized embryos harvested from C57BL/6 mice in the UMMS Transgenic Core to create the endothelial specific PGC-1α transgenic mouse line (PGC-1α EC TG). Genotyping was performed with mouse tail DNA and primers of (ggctggtaccttggaactga) and (aatccgtcttcatccacagg). Two separate PGC-1α EC TG mouse lines were used for experiments. The PGC-1α EC TG mice were bred onto the background of eNOS^−/−^ mice (Jackson Laboratory) to generate PGC1α-EC TG/eNOS^−/−^ mice.

For the endothelial specific knockout line, the PGC-1α allele containing *LoxP* sites flanking exons 3–5 of the PGC-1α gene (*PGC1α*^*flox/flox*^)^32^ was obtained from Bruce Spiegelman (Harvard University) and bred with the Tie2-Cre mouse line on the C57 background. These endothelial-specific PGC-1α knockout mice (PGC-1α EC KO) were compared to Tie2-Cre mice (Cre). Approval for animal care and use for these experiments was granted by the Institutional Animal Care and Use Committee (IACUC) of the University of Massachusetts Medical School and the Ethics Committee of the University Hospital Mainz and all experiments were carried out in accordance with the guidelines from these institutions.

### Cell culture

Primary cultures of human aortic endothelial cells (HAECs) or human umbilical vein endothelial cells (HUVECs) were obtained from Lonza Group Ltd. (Switzerland) and used in experiments during passages 2–8. Cultures were maintained in EBM-2 media with supplements (Lonza). Primary cultures of rat aortic smooth muscle cells (RASMCs) were obtained from VEC Technologies (Rensselaer, NY, USA) and used in experiments during passages 2–8. RASMC cultures were maintained in DMEM (Cellgro) that contained 10% fetal bovine serum, 500 IU/ml penicillin, and 500 IU/ml streptomycin. Mouse lung endothelial cells were prepared by immunoselection with anti-ICAM-2 antibody as previously described^33^. Adenovirus was used for both overexpression (24 h) and knockdown (48 h) of PGC-1α (kind gift from the laboratory of Bruce Spiegelman) and ERRα (kind gift from laboratory of Anastasia Kralli, Scripps Research Institute^34^). Control viruses (siCtl, LacZ, and GFP) from Vector BioLabs (Malvern, PA).

### mRNA extraction and RT-PCR

RNA was extracted from endothelial cells or mice aorta using RNeasy mini kit (Qiagen) or Trizol tissue extraction. For mRNA expression analysis both TaqMan (Life Technologies) and SYBR Green (Bio-Rad) methods were used (Supplementary Table 1). The ΔΔcycle threshold method was used for relative mRNA quantification and the gene expression was normalized to a housekeeping gene (TBP or HPRT).

### Osmotic pump implantation

Mice were anesthetized with an intraperitoneal ketamine-xylazine mixture. Osmotic pumps (Alzet model 1007D) were implanted subcutaneously to allow infusion of ATII or vehicle (NaCl 0.9%) at one of two rates for 7d: i) the suppressor rate of 0.5 mg · kg^–1^ · d^–1^ that does not significantly compromise endothelial function in wild type (WT) mice^35^ or a pressor rate of 1.0 mg · kg^–1^ · d^–1^ for 7 days which induces endothelial dysfunction and hypertension in WT mice^5^.

### Blood pressure

Non-invasive determinations were performed in conscious mice by tail cuff sphygmomanometer using BP-2000 pressure analysis system (Visitech Systems, Apex, NC) which we previously validated against telemetry monitoring^35^. The mice were trained daily for one week prior to recorded measurements. A minimum of 10 preliminary measurements with 20 actual measurements were performed in each session. Measurements were made at the same time each day to account for diurnal variation. Five consecutive daily measurements were averaged for each of the groups. Where indicated, NG-nitro-L-arginine methyl ester (LNAME) was administered (0.5 g/L) in the drinking water for 7–14 days.

### Western blot

The cell and tissue lysates were denatured with Laemmli sample buffer (Cell Signaling) and then resolved by SDS-PAGE followed by western blotting with antibodies as indicated. The protein bands were visualized using the AlphaImager^©^ imaging system and bands quantified with Image J (NIH). Numbers below immunoblots represent densitometry relative to actin.

### Tissue NO• measurement

Electron paramagnetic resonance spectrometry (EPR) was used to assess vascular NO• synthesis using colloid Fe(II)-diethyldithiocarbamate (Fe(DETC)2) as spin trap with an X-band table-top spectrometer MS200 (Magnettech, Berlin, Germany). The instrument settings were: 10 mW microwave power, 0.8 mT amplitude modulation, 100 kHz modulation frequency, 327 mT center field, 10 mT sweep width, 60 s sweep time and 3 scans. Total NO• production was assessed by measurement amplitude of the characteristic triplet EPR signal and expressed in arbitrary units, AU/(mg dry weight × h)^35^.

### Tissue cGMP

Tissue cGMP from aortas was measured as described^33^.

### *In vitro* cGMP

Endothelial NO• bioactivity in culture was estimated as cGMP production in a RASMC reporter assay as previously described^33^.

### Isometric Measurements of Aortic Function

Thoracic aortic rings (2 mm in length) were mounted on 200 μm pins in a 6-mL chamber vessel myograph (Danish Myo Technology; 610 M) with 1 g basal tension as previously described^33^. Aortic rings were subjected to concentration-response curves to increasing concentrations of phenylephrine (PE) and acetylcholine (Ach), the latter in vessels precontracted with a submaximal (70–80%) concentration of PE (10^–7^ M).

### Statistics

Numerical data are presented as mean ± SE. Overall differences were analyzed using one-way or two-way ANOVA and tested with Tukey-Kramer Multiple-Comparison Test for determining differences between the means when more than two groups were compared. An independent *t*-test was used when only two groups were compared. In all tests, significance was accepted at P < 0.05.

## Additional Information

**How to cite this article**: Craige, S. M. *et al*. PGC-1α dictates endothelial function through regulation of eNOS expression. *Sci. Rep.* **6**, 38210; doi: 10.1038/srep38210 (2016).

**Publisher's note:** Springer Nature remains neutral with regard to jurisdictional claims in published maps and institutional affiliations.

## Supplementary Material

Supplementary Information
