# Supplementary material for: PGC-1α dictates endothelial function through regulation of eNOS expression
Source: Sci Rep. 2016 Dec 2;6:38210. doi: 10.1038/srep38210 (PMC5133545; doi:10.1038/srep38210)
Supplement: Supplementary Information [file srep38210-s1.pdf]

CRAIGE et al. SUPPLEMENTAL MATERIAL

PGC-1α dictates endothelial function through regulation of eNOS expression

Siobhan M. Craige[1,2*], Swenja Kröller-Schön[1,3], Chunying Li[2], Shashi Kant[2], Shenghe Cai[2], Kai Chen[3,4], Mayur M. Contractor[2], Yongmei Pei[2], Eberhard Schulz[3,5], John F. Keaney Jr.[2,5, *]

[1] These authors equally contributed
[2] Division of Cardiovascular Medicine, Department of Medicine
University of Massachusetts Medical School
Worcester, MA
[3] Department of Cardiology
Medizinische Klinik und Poliklinik
Universitätsmedizin Mainz
Mainz, Germany.
[4] University of Connecticut Health Center, Farmington, CT
[5] These authors equally contributed
[*]To whom correspondence should be addressed:

Siobhan M. Craige, PhD
Department of Medicine/Cardiovascular Division
University of Massachusetts Medical School
Worcester, MA 01655, USA
Phone: 508-856-6904
Fax: 508-856-6933
Email: Siobhan.craige@umassmed.edu

John F. Keaney, Jr., MD
Department of Medicine/Cardiovascular Division
University of Massachusetts Medical School
Worcester, MA 01655, USA
Phone: 508-856-6901
Fax: 508-856-6933
Email: John.keaney@umassmed.edu

Supplemental Methods:

Materials: Antibodies: Akt, pAkt (Cell Signaling)

Mitochndrial ROS production:
Wild-type and PGC-1α EC TG MLECs were incubated with 200 nM MitoTracker Green FM (MitoG) for 50 min and subsequently 5 μM MitoSOX Red for 25 min. MitoSOX and MitoG signals were measured respectively on a fluorescence plate reader (MitoSOX: Ex = 396 or 510 nm, Em = 580 nm; MitoG: Ex = 490 nm, Em = 516 nm).

Craige et al.
Supplementary Figure 1

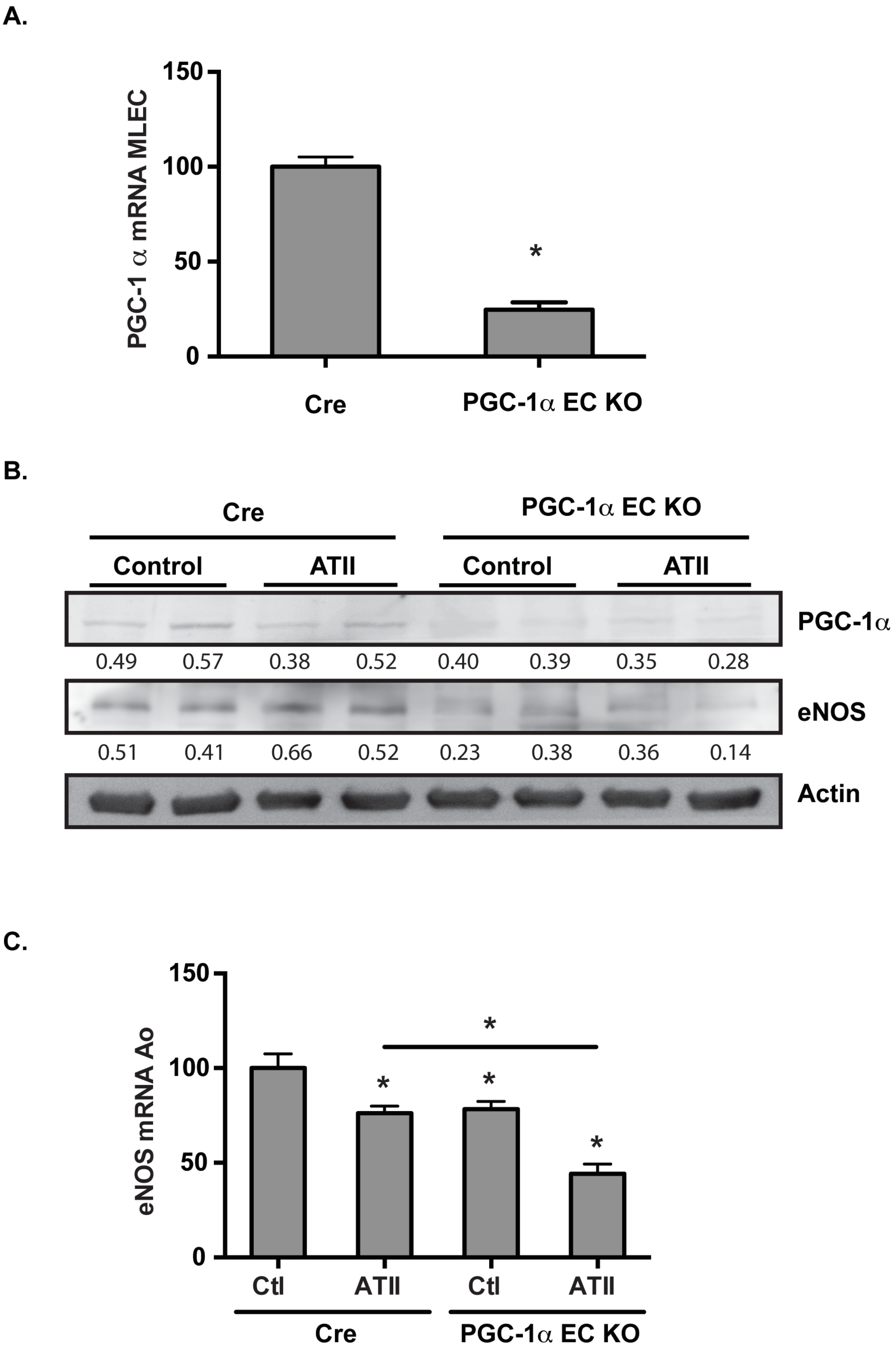

Craige et al.
Supplementary Figure 2

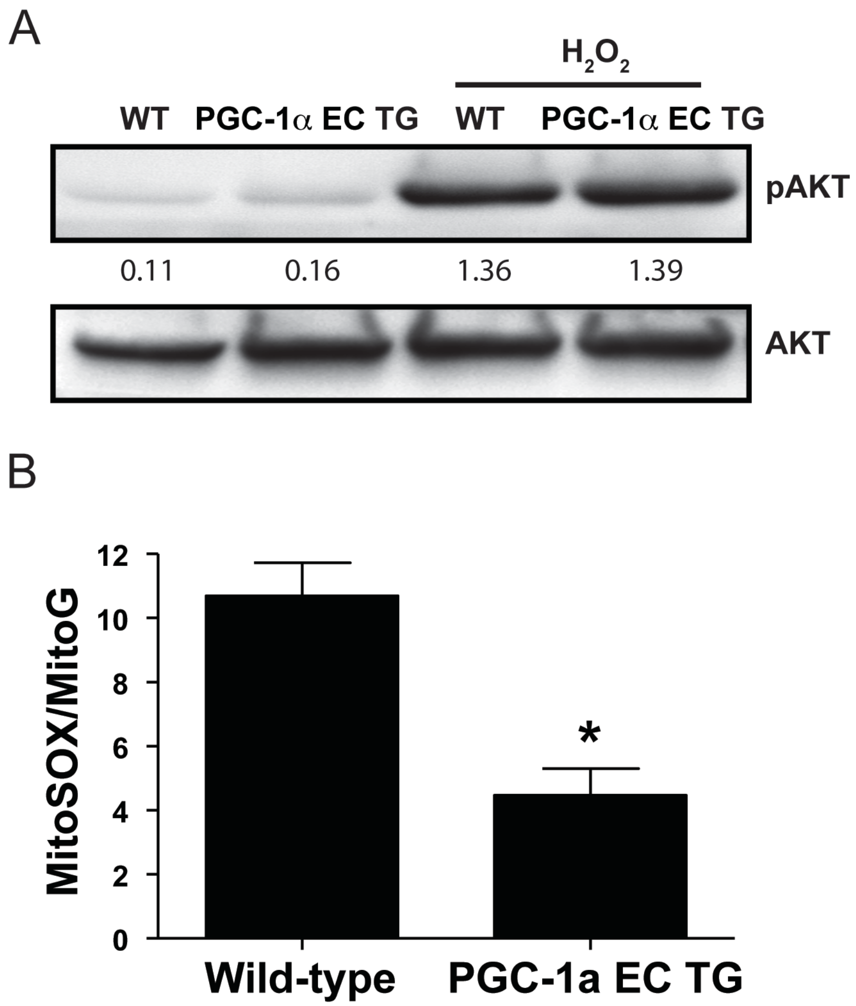

Supplemental Figure Legends:

Supplemental Figure 1:

(**A**) MLEC were harvested from PGC-1α EC KO and Cre (control) mice and PGC-1α expression measured. (N = 6/ group *P < 0.05) (**B**) Mice were treated with subpressor ATII (0.5 mg/kg; 7 days) and aorta were harvested from PGC-1α EC KO and Cre mice for immunoblotting. (N = 8/group; *P < 0.05) (**C**) Aorta were harvested from mice as in (B) and eNOS expression measured. (N = 6/group; *P < 0.05 vs. Cre Ctl; #P < 0.05 vs Cre ATII)

Supplemental Figure 2:

(**A**) MLEC were harvested from WT and PGC-1α EC TG mice and immunoblotted for Akt expression and activation +/- H_2O_2. (**B**) MLEC were harvested from WT and PGC-1α EC TG mice and mitochondrial ROS were measured (N = 6; *P < 0.05).

Supplemental Table 1: Gene expression primer list

Taqman probes:
TATA box binding protein (TBP; Mm00446973_m1)
PGC-1α (Mm01208835_m1)
eNOS (Mm00435204_m1)
ERRα (Mm00433143_m1)
SYBR Green primers:
mouse:
PGC-1α F: GCCGTGTGATTTACGTTGGTAA, R: AAAACTTCAAAGCGGTCTCTCAA
eNOS F: GACCCTCACCGCTACAACAT, R: CTGGCCTTCTGCTCATTTTC
ERRα F: GCAGGGCAGTGGGAAGCTA, R: CCTCTTGAAGAAGGCTTTGCA
HPRT F: TGGCCATCTGCCTAGTAAAGC, R: GGCTCATAGTGCAAATCAAAAGTC;
TBP F: ACCCTTCACCAATGACTCCTATG, R: TGACTGCAGCAAATCGCTTGG.
human:
PGC-1α F: TGTCACCACCCAAATCCTTATTT, R: TGTGTCGAGAAAAGGACCTTGA
eNOS F: AGGAACCTGTGTGACCCTCA, R: CGAGGTGGTCCGGGTATCC
ERRα F: AAGACAGCAGGCCCAGTGAA, R: ACACCCAGCACCAGCACC
HPRT F: GCTCGAGATGTGATGAAGGAGAT, R: AGGTCAGCAAAGAATTTATAGC
TBP F: GCACAGGAGGCCAAGAGTGAA, R: TCACAGCTCCCCACCATGTT
